# Transcriptome analysis during seed germination of elite Chinese bread wheat cultivar Jimai 20

**DOI:** 10.1186/1471-2229-14-20

**Published:** 2014-01-13

**Authors:** Yonglong Yu, Guangfang Guo, Dongwen Lv, Yingkao Hu, Jiarui Li, Xiaohui Li, Yueming Yan

**Affiliations:** 1 College of Life Science, Capital Normal University, Beijing 100048, China; 2 Department of Plant Pathology, Kansas State University, Manhattan KS 66506, USA

**Keywords:** Bread wheat, Seed germination, Transcriptome, qRT-PCR

## Abstract

**Background:**

Wheat seed germination directly affects wheat yield and quality. Although transcriptome and proteome analyses during seed germination have been reported in some crop plant species, dynamic transcriptome characterization during wheat seed germination has not been conducted. We performed the first comprehensive dynamic transcriptome analysis during different seed germination stages of elite Chinese bread wheat cultivar Jimai 20 using the Affymetrix Wheat Genome Array.

**Results:**

A total of 61,703 probe sets representing 51,411 transcripts were identified during the five seed germination stages of Jimai 20, of which 2,825 differential expression probe sets corresponding to 2,646 transcripts with different functions were declared by ANOVA and a randomized variance model. The seed germination process included a rapid initial uptake phase (0–12 hours after imbibition [HAI]), a plateau phase (12–24 HAI), and a further water uptake phase (24–48 HAI), corresponding to switches from the degradation of small-molecule sucrose to the metabolism of three major nutrients and to photosynthesis. Hierarchical cluster and MapMan analyses revealed changes in several significant metabolism pathways during seed germination as well as related functional groups. The signal pathway networks constructed with KEGG showed three important genes encoding the phosphofructokinase family protein, with fructose-1, 6-bisphosphatase, and UTP-glucose-1-phosphate uridylyltransferase located at the center, indicating their pivotal roles in the glycolytic pathway, gluconeogenesis, and glycogenesis, respectively. Several significant pathways were selected to establish a metabolic pathway network according to their degree value, which allowed us to find the pathways vital to seed germination. Furthermore, 51 genes involved in transport, signaling pathway, development, lipid metabolism, defense response, nitrogen metabolism, and transcription regulation were analyzed by gene co-expression network with a k-core algorithm to determine which play pivotal roles in germination. Twenty-three meaningful genes were found, and quantitative RT-PCR analysis validated the expression patterns of 12 significant genes.

**Conclusions:**

Wheat seed germination comprises three distinct phases and includes complicated regulation networks involving a large number of genes. These genes belong to many functional groups, and their co-regulations guarantee regular germination. Our results provide new insight into metabolic changes during seed germination and interactions between some significant genes.

## Background

Wheat (*Triticum aestivum* L., 2n = 6x = 42, AABBDD), an allohexaploid species, is one of the most important and widely cultivated crops in the world. Wheat has extensive agronomic adaptability and can be cultivated from 67° N in Scandinavia and Russia to 45° S in Argentina, including the tropics and subtropics [1,2]. The crop is used largely for human food and livestock feed.

The chemical components of wheat seed include protein, carbohydrate, lipid, nucleic acid, pigment, vitamin, enzymes, and inorganic materials. Mature wheat seeds consist mainly of starch (up to 70%) and proteins (12–15%). Carbohydrates exist mainly in the form of starch, which accounts for 55–70% of the whole seed weight and is activated to provide energy when the seeds germinate. Seed proteins include albumins, globulins, gliadins, and glutenins [3,4], of which gliadins and glutenins are the main storage proteins that determine the viscoelasticity of dough [5]. Their mobilization also provides energy and other intermediate products during seed germination. Albumins and globulins are important in human nutrition because they are abundant in essential amino acids such as lysine, tryptophan, and methionine [5].

Seed germination commences with imbibition, the uptake of water by the quiescent dry seed, and terminates with the elongation of the embryonic axis [6]. Subsequent reserve mobilizations are associated with seedling growth. The seed undergoes a three-phase process of physiological and morphological changes. The first phase (phase I) is a rapid initial uptake, in which seeds begin to expand and the seed coat becomes softer. Meanwhile, the physical state of storage materials such as starch, proteins, and lipids change gradually. Then the germinated seed enters a plateau phase (phase II) and water uptake increases (phase III). The third phase occurs as the embryonic axes elongate after germination is complete [7]. Respiration and energy production play key roles in whole seed germination. In the beginning, the energy for seed germination is mainly provided by anaerobic respiration, then respiratory activity increases as oxygen uptake and carbon dioxide release accelerate during imbibition. Oxygen uptake is associated with oxidation phosphorylation through cytochrome oxidase [8].

Because seed germination is highly related to seedling survival rate and subsequent vegetative growth, it directly affects wheat yield and quality. Initial primary studies on seed germination focused mainly on aspects of seed physiology and biochemistry [6,7,9,10] and allowed us to have a basic understanding of the seed germination process. In recent years, proteome and transcriptome approaches have been used to study the biochemical mechanisms of plant seed germination. Considerable proteomics work has been performed on the studies of seed germination in many plants, such as *Arabidopsis*[11], cress [12], sugar beet [13], *Medicago truncatula*[14], barley [15], maize [16] and rice [17]; however, the number of proteins detected by the proteome approach is limited and therefore does not allow a complete genome-wide comparison. Dry mature seeds contain a large number of mRNA species. Cotton was the first plant found to store RNA in a mature dry seed [18]; after the 1990s, stored RNA was found to be universal in the mature dry seeds of plant species [19-21]. Stored mRNA in seeds reflects gene expression patterns during seed germination [22]. Affymetrix arrays can provide a comprehensive description and real-time changes at the whole-transcriptome level during seed germination and have been used to investigate the biological processes of seed germination in many plants such as Arabidopsis [22], barley [23,24], rice [25] and maize [26]. Wheat grain development has been studied mostly by transcriptome approaches [27-29]. Investigations into wheat seed germination at the transcriptional level have remained limited to a particular germination stage [30]; comprehensive dynamic transcriptome characterization during wheat seed germination has not been done.

Modern allohexaploid wheat with A, B, and D genomes have a huge and complex genome (up to 17,000 Mb). Wheat genome sequencing has made recent progress [31]. Genome projects on A^u^ genome in *Triticum urartu* and D^t^ genome in *Aegilops tauschii*, the progenitors of A and B genomes in hexaploid wheat, respectively, have been completed [32,33] and have facilitated further proteome and transcriptome studies on wheat seed germination. In this study, we used Jimai 20, an elite Chinese bread wheat cultivar with high yield, wide adaptability, and superior quality [34], to perform the first dynamic transcritpome microarray analysis during five germination stages using the GeneChip® Wheat Genome Array (Affymetrix, Santa Clara, CA). Our results provide new insights into the molecular mechanisms of wheat seed germination.

## Results

### Morphology and SEM observation during seed germination progress

**Figure 1:**
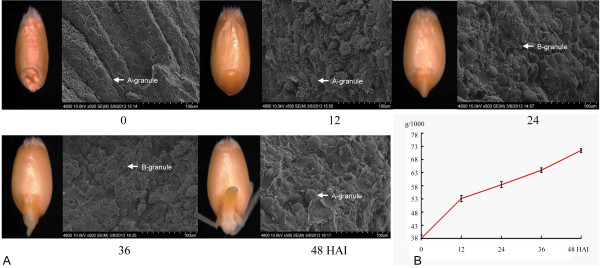
**Five phases of grain germination used for transcriptome analysis. A**, Grain appearance and the structural changes of wheat endosperm based on SEM. A- and B-granules are indicated. **B**, Changes in 1,000-seed weight during the germination process.

Upon imbibition, wheat seed inflated gradually, but embryo appearance was relatively unchanged in the first 12 hours after imbibition (HAI), as shown in Figure 1A. At 24 HAI, the structures surrounding the embryo were penetrated by the radical, and both radical and bud emerged at 36 HAI. The plumule become slightly green and radicals elongated at 48 HAI.

During the germination process, seed weight increased with imbibition (Figure 1B). Water uptake is rapid in the early germination stage; consequently, seed weight increased rapidly at 12 HAI and continued to increase steadily from 24 to 48 HAI. Abundant early imbibition establishes a basis for the mobilization of water-soluble metabolites, which provide the continuous energy for the seed germination and subsequent seedling growth.

Wheat endosperm contains three kinds of starch granules before imbibition: A-type (diameter more than 10 μm), B-type (5–10 μm) and C-type (less than 5 μm). These starch granules provide important energy supplies for seed germination. Scanning electronic microscope (SEM) observation of endosperm demonstrated that the diameter of A-type starch granules increased along with seed imbibition (Figure 1A and Additional file 1: Figure S1). Endosperm space become sparse after water uptake, and almost all starch granules inflated, indicating that storage starch gradually degraded to provide energy for germination.

### Transcriptome expression profile and functional categories during seed germination

To investigate transcriptome expression profiles during seed germination, we performed a gene chip analysis with GeneChip® Wheat Genome Arrays, which contain 61,127 probe sets representing 55,052 transcripts [35]. In our experiment, a total of 61,703 probe sets (the sum of 61,115 actual probe sets and the other 588 repeated probe sets) representing 51,411 transcripts were identified (Additional file 2: Table S1). The 61,703 probe sets can be classified into 35 BINs; among 19,782 probe sets with different functions, 34 BINs were classified with automatic annotations of metabolic pathways and large enzyme families. Another 41,921 probe sets were classified as unknown or not assigned (Additional file 2: Table S2). The probe sets related to protein, RNA, enzyme families, signaling and transporters were a large proportion of those identified with functions, indicating their significant roles during seed germination.

**Figure 2:**
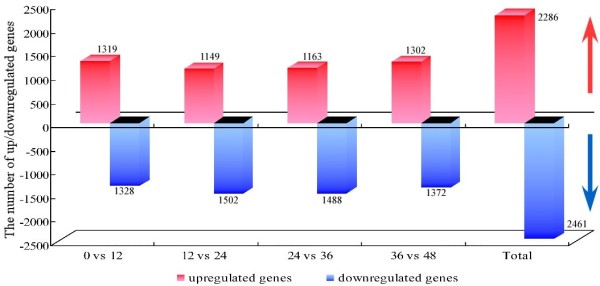
**The number of up/downregulated genes during five seed germination stages.** Each group consists of a red column (the number of upregulated genes) and blue column (the number of downregulated genes). The numeral over the column is the number of up/downregulated genes. The former four groups are the number of genes between successive time points, and the last group is the total number of genes during the seed germination process.

Genome-wide transcriptional profiling demonstrated that extensive gene expression occurred during germination. A total of 2,825 probe sets corresponding to 2,646 transcripts were declared differentially expressed during five seed germination phases (Additional file 2: Table S3). When successive time points were compared, the number of up- or downregulated genes between two successive time points was obtained (Figure 2). Results showed that 2,286 genes were upregulated, whereas 2,461 genes were downregulated during the seed germination process.

**Figure 3:**
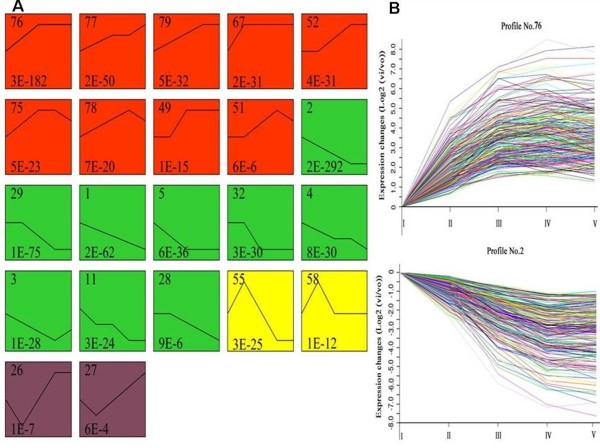
**The main expression patterns of 2,646 transcripts analyzed by model profile and the two significant patterns (profile No. 2 and No. 76). A**, Each box represents a model expression profile. The upper number in the profile box is the model profile number, and the lower one is the *p*-value used to summarize the different gene expression patterns. In total, 22 expression patterns of genes showed significant *p*-values (*p* < 0.05). The same color represents the same group. The model profiles marked with red (group I) represent the upregulated expression patterns, whereas the green profile boxes (group II) represent the downregulated expression patterns. Profile No. 55 and 58 (group III) belong to the pattern that was upregulated first then downregulated, whereas the remaining profile boxes (group IV) belong to the opposite pattern with group III. **B**, Profile No. 2 increased in expression and profile No. 76 decreased in expression during seed germination. The horizontal axis represents the germination phase, and the vertical axis shows the time series of gene expression levels for the gene after Log2 normalized transformation.

Further analysis of 2,646 transcripts demonstrated that 22 expression profiles were highly significant, and these profiles were classified into four groups. As shown in Figure 3, the upregulated genes in group I and downregulated genes in group II accounted for 87.7% of the total differentially expressed genes. Genes in group III displayed upregulated expression between 0 and 12 HAI but were downregulated sharply during the late germination progress. The genes in group IV were initially downregulated, then upregulated gradually after 12 HAI. Among 22 expression profiles, the two most significant patterns (profiles No. 2 and No. 76) according to their *P*-values had the largest number of differentially expressed genes, including 239 and 245 genes, respectively (Figure 3).

**Figure 4:**
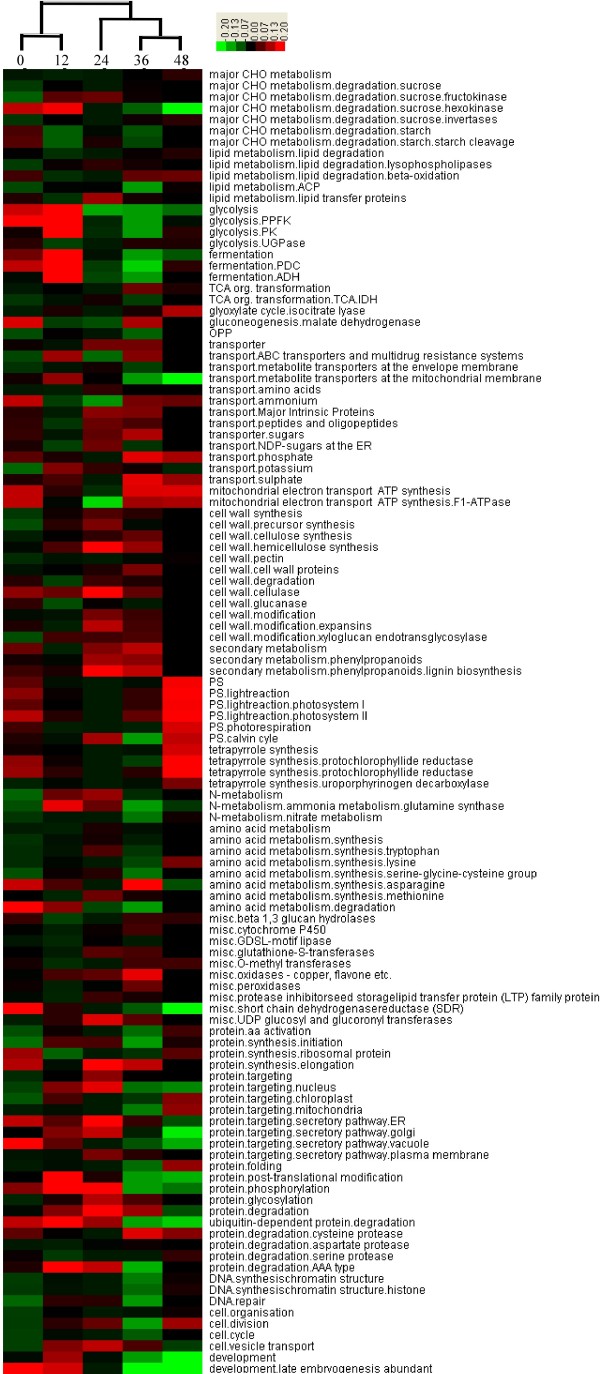
**Hierarchical cluster analysis (HCA) revealed the coordinated changes of important gene functional categories activated during seed germination phases (0/12/24/36/48 HAI).** The color scale is: red, high expression; black, moderate expression; green, low expression.

Hierarchical cluster analysis (HCA) was used to reveal the coordinate changes in some important gene functional categories that were activated during each phase of seed germination (Figure 4). Three distinct phases corresponding to two clear switch points at 12 and 24 HAI could be divided according to cluster. The first phase with rapid initial uptake was 0–12 HAI, when genes involved in small-molecule sucrose degradation were activated. The second phase was from 12–24 HAI, when metabolism genes related to three major nutriments (carbohydrates, proteins and lipids) and cell wall metabolism began to be activated. From 24 to 36 HAI, seed germination entered the third phase, a distinct transition stage in which the metabolic genes were continuously and abundantly expressed. At the end of germination (48 HAI), genes related to photosynthesis were activated and the embryonic axis clearly elongated. Thus, the seed germination process underwent two main switches, from the degradation of small-molecule sucrose to the metabolism of three major nutriments and then to photosynthesis. Consequently, energy reserve decreased throughout the germination process, especially at 24 and 36 HAI.

### Energy provision from reserve mobilization

**Figure 5:**
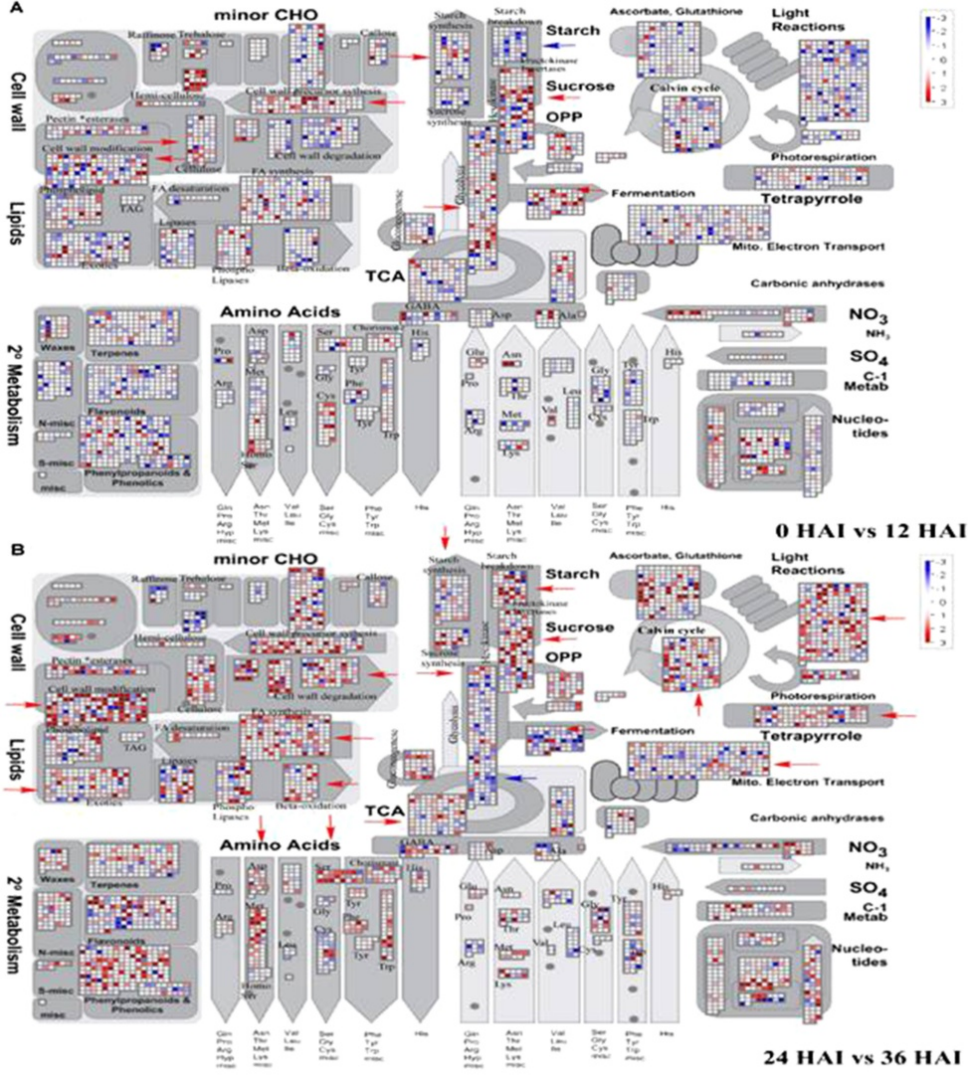
**MapMan metabolism overview maps showing differences in transcript levels (0 versus 12 and 24 versus 36 HAI) during seed germination. A**, 0 vs. 12 HAI. **B**, 24 vs. 36 HAI. Log2 ratios for average transcript abundance were based on three replicates of Affymetrix GeneChip® Wheat Genome Array. The resulting file was loaded into the MapMan Image Annotator module to generate the metabolism overview map. On the logarithmic color scale, blue represents downregulated transcripts, and red represents upregulated transcripts.

MapMan has been shown to be an effective tool to map transcriptome data, define functional categories, and perform time course analyses for identifying significantly overrepresented functional groups and has been applied to transcriptome analysis in Arabidopsis, barley and rice [24,25,36]. In this study, important gene functional groups activated in different seed germination stages were analyzed by MapMan, and the results are shown in Figure 5 and Additional file 2: Table S2.

Reserve accumulation in the endosperm is initiated in early seed development then ends until the seed matures. During maturation, wheat seeds accumulate reserve materials, including starch, sucrose, lipids, and storage proteins. Metabolic activities within the seed are drastically downregulated during dormancy then reactivated during imbibition, germination, and seedling growth. When seeds began to imbibe water, dormancy is broken and the quiescent embryo is able to germinate. In addition to the mobilization of reserves, an important stage for shoot apical meristem (SAM) and root apical meristem (RAM) development occurs during seed germination. Several development-related genes were found to be significantly upregulated at 24 HAI, such as CK202188 and BJ272365 related to the WUSCHEL-CLAVATA pathway and CD869453 and CK201893 related to SAM and RAM (Table 1). In addition, some meristem-related genes encoding signaling receptor kinases, such as BJ266952 and BJ307684, also showed upregulated expression patterns during seed germination.

**Table 1:** The functional annotation and expression values of some important genes

**Genes**	**Gene functional annotation**	**Expression values**				
		**0 HAI**	**12HAI**	**24 HAI**	**36 HAI**	**48 HAI**
CK202188	Signaling (CLV1)	169.4	233.8	739.1	642.5	920.4
BJ272365	Development (WUS)	855	1327.5	1869.6	1641.7	2129
CK201893	Redox (RML1)	1197.5	3513.7	4029.8	6636.6	7191.3
CD869453	RNA (STM)	78.1	171.6	701.5	931.5	512.9
CD866884	PPFK	2402.5	16708	4057.1	3532	4693.6
BJ252827	PK	874.9	12434.8	2758.3	2533.1	2731.5
CK207050	PK	817.4	12462.8	3732.2	3005.6	2940.7
CD491538	UGPase	95.6	65.1	340.7	416.1	464.5
CD868238	ADH	46.5	18628.9	7036.5	6872.1	11337.4
CD934753	PDC	26377	29397.3	24081.2	21766.5	22919.1
CA719001	AMY1/ATAMY1	216.9	107.8	11673.8	17917.4	15255.9
AL820663	AMY1/ATAMY1	436.2	187.7	16939.6	22679.4	16296.5
BF293263	AMY1/ATAMY1	90.1	19.8	4794.2	10521.7	3553.7
CD490513	Embryonic abundant protein 1	8096.9	3918.1	411.2	11	15
CD452864	CAX3 (cation exchanger 3)	3725.7	3014.4	1492.8	1087.4	1101.8
CK208119	CYP78A8 (cytochrome P450, family78, subfamilyA, polypeptide 8)	1162.8	600.6	242.6	173.6	154.8
CK216168	Ethylene forming enzyme	131.1	1223.2	2211.7	3156.8	2767.1
BJ291458	UBQ3 (Polyubiquitin 3)	1894.2	698.1	261.5	124.8	141.7
BJ282439	Ubiquitin E3	2361.7	2223.4	1081.8	906.4	767.7
CD453856	Chaperonin; folding	560.8	1558.8	3795.8	3980.4	3377.9
CD373830	Zinc transporter	1018.6	1032.4	627.1	505.7	450.9
CA649522	Posttranslational modification	1288.8	2373.7	5361.1	7029.1	5254.5
M26672.1	Susy (sucrose synthase)	53.8	74.1	131.5	120.4	169.8

According to gene expression data, mobilization of deposited starch, sucrose, and storage proteins begins early in seed germination (Figure 5A and Additional file 3: Figure S2A). For example, glycolysis was activated at the beginning of imbibition along with the mobilization of accumulated sucrose (Figures 4 and 5). In this process, genes encoding related key enzymes such as sucrose synthase (SUSY), hexokinase, phosphofructokinase (PPFK), and pyruvate kinase (PK), which promote glycolysis, all displayed upregulated expression (Figures 4 and 5A). For instance, from 0 to 12 HAI, as the key genes in glycolysis, the gene CD866884 encoding PPFK had 7-fold upregulation, whereas BJ252827 and CK207050 encoding PK displayed 14- and 15-fold upregulation, respectively (Table 1); however, the gene (CD491538) encoding UDP-glucose pyrophosphorylase (UGPase), the key enzyme for starch synthesis, was downregulated in glycolysis (Table 1 and Figure 4). Correspondingly, due to the continuous synthesis of pyruvate, which then turned into alcohol and entered the fermentation pathway, the genes involved in the fermentation process were upregulated as glycolysis. For example, two important enzymes of catalysis in fermentation, pyruvate decarboxylase (PDC, CD934753) and alcohol dehydrogenase (ADH, CD868238), were significantly upregulated (Figure 4 and Table 1). Both glycolysis and fermentation pathway–related genes were downregulated after 12 HAI, probably due to the end of the mobilization of stored sucrose (Figures 4 and 5A).

Seed germination then entered the stationary phase. Starch mobilization occurred after sucrose degradation. When the seed endosperm imbibed a certain amount of water, the starch genes were activated in the endosperm between 12 and 24 HAI and reached a period of great prosperity after 24 HAI, then related genes were gradually downregulated after 36 HAI due to the continuous consumption of starch (Figure 5B and Additional file 3: Figure S2).

Amylases are major enzymes for wheat starch cleavage. In our study, most genes related to alpha-amylase, such as CA719001, AL820663 and BF293263, were initially upregulated starting at 12 HAI and reached a maximum at 37 HAI, whereas expressions of beta-amylase were downregulated during the seed germination process (Table 1). This result indicates that alpha-amylase is a major enzyme that hydrolyzes starch for glucose provision. Along with the transcriptional activation of starch-degrading genes, the transcripts of genes encoding fructokinase invertases and hexokinase, sucrose synthesis and glycolysis increased (Figure 5B and Additional file 3: Figure S2B), which might be due to hexose supply and sucrose synthesis from mobilized starch reserves [24].

The storage of organic material from seed endosperm or cotyledon should be broken down into small molecular compounds such as ammonium, phosphate, amino acids, peptides, oligopeptides, sugars, and major intrinsic proteins and transported to the radicle and embryo in the process of use, so the transport genes were activated and upregulated following the reserve mobilization (Figure 4). Our results showed that the transcripts encoding metabolite transporters at the mitochondrial membrane accumulated most quickly from 0 to 12 HAI, whereas the transcripts encoding metabolite transporters at the envelope membrane were upregulated most quickly from 12 to 24 HAI (Figure 4).

Lipid mobilization begins soon after imbibition, followed by a lag phase. Lipase is a key enzyme for lipid hydrolysis. As a result, fatty acid (FA) is synthesized continuously (Figure 5A and Additional file 3: Figure S2A). After 24 HAI, the transcripts of FA synthesis were highly upregulated, and their expression was gradually downregulated at the end of lipid mobilization (Figure 5B and Additional file 3: Figure S2B). Genes involved in the β-oxidation pathway as a major pathway of FA degradation and phospholipases also were significantly upregulated from 24 to 36 HAI (Figure 5B). β-oxidation is involved in lipid degradation, which eventually results in energy release by peroxisomes and provides a carbon supply for the production of sucrose [24].

Storage proteins are located mainly in the endosperm and aleurone. According to our gene expression data, mobilization of deposited storage proteins begins during late seed germination (Figure 5B), and their metabolic products, amino acids, serve as important nutritional resources for early seedling growth after germination. During this and the following period (from about 36 HAI), the transcripts for storage protein degradation, such as cysteine protease, aspartate protease and serine protease, increased significantly (Figure 4). Genes for amino acid metabolism, such as Asp, Met, Ser, Cys, Phe and Trp, were simultaneously activated (Figure 5B and Additional file 3: Figure S2B). From 12 to 24 HAI, the gene expression related to protein modifications such as phosphorylation and glycosylation increased (Figure 4). This result suggests that synthesis of some clastic enzymes is necessary to mobilize storage protein at this germination stage.

According to our experiments, photosynthesis genes were mobilized slightly after initiation of reserve mobilization during seed germination. As shown in Figure 1A, the cotyledons gradually became green at 48 HAI, indicating that preparations for photosynthesis were almost finished. The genes for photosynthesis, including those related to light reaction, photorespiration, and the Calvin Cycle, were activated, and their expression was highly upregulated at the end of 36 HAI (Figures 4 and 5B).

The seeds became tumescent quickly due to water absorption at the beginning of germination. Genes related to the major constituents of the cell wall (cellulose, pectin, hemi-cellulose, expansins) were activated at 0 to 12 HAI (Figures 4 and 5A). Due to the stasis following imbibition, the related genes of cell wall synthase and degradation were almost downregulated. The seed does not take up water again until the radicle breaks through the seed coat, then these genes are gradually upregulated (Figure 5B and Additional file 3: Figure S2A). The cell wall is largely degraded after 24 HAI (Figure 5B), which activates some important enzymes related to degradation, such as cellulases and pectate lyases (Additional file 2: Table S1). Meanwhile, the genes for cell wall synthesis and modification were largely upregulated (Figures 4 and 5B). This indicates that lots of fractured cell wall was degraded into small molecules to provide raw materials for cell wall synthesis during seed germination.

### Signal pathway network analysis

**Figure 6:**
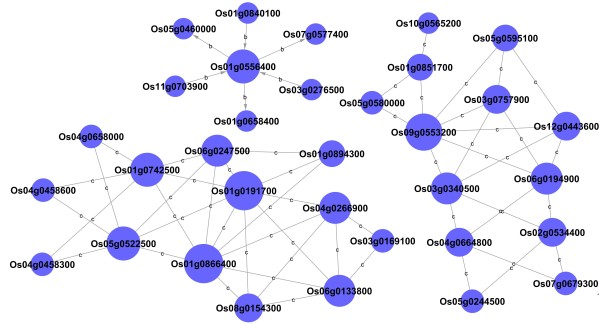
**Signal pathway-net established according to the signal genes functions by Java in the KEGG database.** Cycle nodes represent genes. The size of nodes represents the power of the interrelation among the nodes by degree value. Edges between two nodes represent interactions between signal genes. The letter b means binding or association interaction, and letter c represent compound interaction between two genes.

A signal pathway network was established according to the functions of signal genes by Java in the Kyoto Encyclopedia of Genes and Genomes (KEGG) database (Figure 6). In the network, three genes are at the center of a signal pathway network: Os01g0191700 (phosphofructokinase family protein), Os01g0866400 (fructose-1, 6-bisphosphatase), and Os09g0553200 (UTP-glucose-1-phosphate uridylyltransferase) (Additional file 4: Table S4 and Table S5). Phosphofructokinase is a rate-limiting enzyme in the glycolytic pathway that provides a phosphoryl group from ATP to facilitate a wide variety of biological processes. The expression pattern of PPFK family protein belonged to profile No. 55, which was upregulated at first then sharply downregulated after 12 HAI. Fructose-1, 6-bisphosphatase is an enzyme that converts fructose-1, 6-bisphosphate to fructose-6-phosphate in gluconeogenesis. Its transcriptional level placed it in profile No. 27, which trended opposite the PPFK family protein. Activation of fructose-1, 6-bisphosphatase in gluconeogenesis seems to play an important role in amino acid metabolism. UTP-glucose-1-phosphate uridylyltransferase, also known as UTP-glucose pyrophosphorylase (UGPase), is associated with glycogenesis, which synthesizes UDP-glucose from glucose-1-phosphate and UTP and was upregulated throughout germination, reaching its peak at 36 HAI.

### Metabolic pathway network analysis

**Figure 7:**
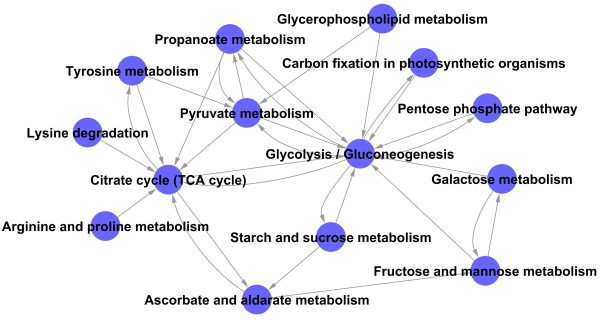
**Metabolic pathway-net established with significant pathways.** Cycle nodes represent different pathways. Arrows represent the interaction between one pathway and another pathway by degree value. The source and point of the arrow show the source of the pathway and the target of the pathway in the network. The node with more arrows means more pathways have interaction with this one, which shows that the pathway is more important in the pathway-net.

Metabolic pathway network was established as shown in Figure 7. According to the degree value (Additional file 5: Table S6), glycolysis and gluconeogenesis metabolisms were located at the center of pathway net, suggesting that the genes involved in glycolysis and gluconeogenesis such as those encoding the PPFK family protein, PK and fructose-1, and 6-bisphosphatase, play key roles in providing energy for seed germination. The citrate cycle (TCA cycle) had the second-highest degree value in the pathway net and mainly provided energy for the late germination of seeds. As the key enzymes of the TCA cycle, 4 enzyme-coding genes (isocitrate dehydrogenase family protein, pyruvate dehydrogenase E1 component beta subunit, malate dehydrogenase and dihydrolipoyllysine-residue succinyltransferase component of 2-oxoglutarate dehydrogenase complex) were upregulated gradually during germination. These genes related to energy metabolism were initiated early in the process, indicating the activation of respiratory activities in mitochondria and ATP release. In accordance, at least 10 genes related to vacuolar ATPase and mitochondrial ATP synthase complexes and mitochondrial carrier proteins were preferentially upregulated during seed germination (Additional file 2: Table S1). The alcohol dehydrogenase (ADH) gene in the fermentation pathway was also upregulated sharply to provide energy for seed germination. Pyruvate is an important intermediate product that plays a mediated role, and pyruvate metabolism is another important pathway. Pyruvate metabolism converts pyruvate into acetyl-CoA, which is used in lipid biosynthesis and the TCA cycle, in two ways [17,37]. One way is catalyzed by pyruvate dehydrogenase (PDH), which catalyzes the conversion of pyruvate to acetyl-CoA. The other way is catalyzed by pyruvate decarboxylase (PDC) and aldehyde dehydrogenase (ALDH), which catalyzes the irreversible conversion of pyruvate to acetaldehyde and CO_2_, so acetaldehyde can be converted into acetate to produce additional acetyl-CoA.

From the metabolic pathway network, many amino acids, including lysine, tyrosine, arginine and praline, were triggered to provide a major nitrogen source for nitrogen metabolism and intermediates for the TCA cycle. Three kinds of enzymes are involved in lysine degradation: the aldehyde dehydrogenase (ALDH) family protein, a SET domain containing protein and the dihydrolipoyllysine-residue succinyltransferase component of the 2-oxoglutarate dehydrogenase complex. Fumarylacetoacetase, aspartate aminotransferase and alcohol dehydrogenase 2 are involved in tyrosine metabolism. In arginine and proline metabolism, the expression of four enzyme genes (aldehyde dehydrogenase, aspartate aminotransferase, delta-1-pyrroline-5-carboxylate synthetase and argininosuccinate lyase) was upregulated compared with the dry seed, whereas two enzyme genes (aldehyde dehydrogenase family protein and glutamine synthetase root isozyme 2) were gradually downregulated during germination (Additional file 2: Table S1).

### Gene co-expression network with k-core algorithm

**Figure 8:**
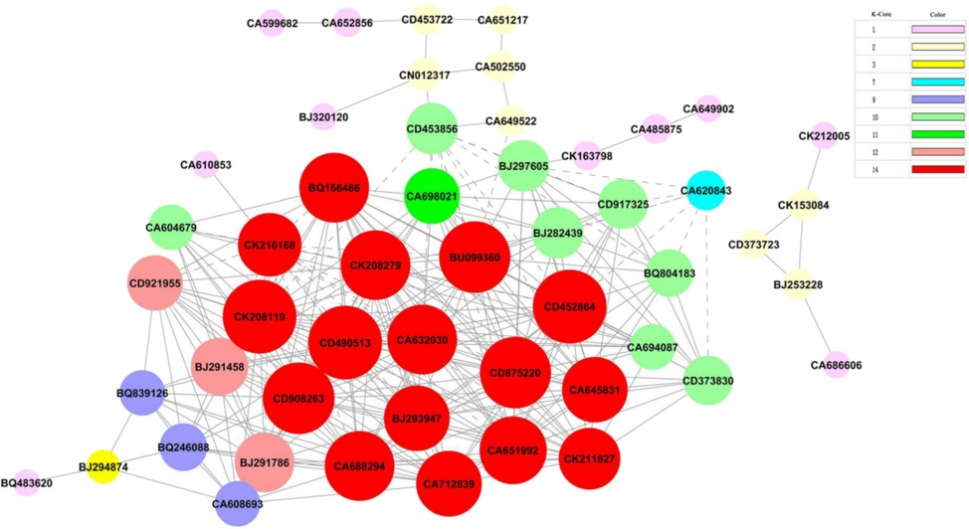
**Gene co-expression network during seed germination with k-core algorithm.** Cycle nodes represent genes, the size of nodes represents the power of the interrelation among the nodes, and edges between two nodes represent interactions between genes. The more edges on a gene, the more genes connect to it, and the more central role it has within the network. In the network, the solid line is positive regulation and the dashed line is negative regulation.

To determine the functional genes that play pivotal roles in the germination of wheat seed, genes involved in some important pathways were further analyzed by gene co-expression network with a k-core algorithm (Figure 8). The most central gene has the highest degree value within the network. According to k-core values, 31 genes with higher k-core levels in our results were considered to have a core status (Additional file 6: Table S7). Most of the genes are attributed to transport, signaling pathway, development, lipid metabolism, stress and defense response, nitrogen metabolism and the transcription progress. Three of the most important core genes, CD490513 encoding embryonic abundant protein 1, CD452864 encoding CAX3 (cation exchanger 3), and CK208119 encoding CYP78A8, were located at the center of the network with the highest k-core levels. They directly regulated 24 neighboring genes according to their degrees (Additional file 6: Table S7). These genes decreased drastically during germination, especially CD490513 (Table 1). In the network, CK216168 (ethylene-forming enzyme) is one of the upregulated genes (Table 1) and is located at the center of network with k-cores of 14 and 19 degrees, indicating its involvement in a variety of functions including defense response, signal transduction, lipid metabolic process, the carbohydrate metabolic process and the ethylene biosynthetic process.

In the network, three genes, BJ291458, BJ282439 and CD453856, were involved in protein metabolism. BJ291458 (UBQ3), with k-cores of 12 and 16 degrees, and BJ282439 (Ubiquitin E3), with k-cores of 10 and 13 degrees, are mainly involved in regulating the degradation of proteins (Table 1). Because the products of protein degradation need be transported from endosperm to embryo, co-adjustment between the related genes of protein degradation and the correlative genes of transport (CD452864 and CD373830) are consistent and show that these genes are positively regulated in the network, suggesting that the down/upregulation of protein degradation genes would lead to the down/upregulation of transport genes. The function of CD453856 (Chaperonin; folding) is to regulate protein folding, then catalyze the modification of protein by regulating the other gene that participates in the posttranslational modification, CA649522 (Figure 8 and Table 1). Because the function of both CD453856 and CA649522 is the process opposite protein degradation, we can infer that the co-adjustment between CD453856 and CD452864 is negative regulation, which is consistent with the results shown in Figure 8.

### Verification of gene expression patterns by qRT-PCR

**Figure 9:**
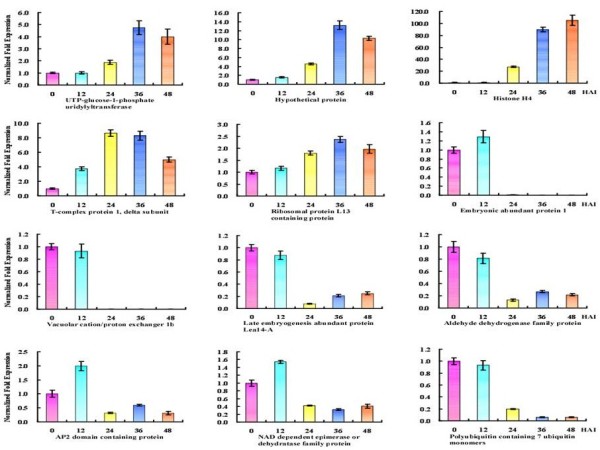
**Verification of 12-gene expression patterns by qRT-PCR.** The horizontal axis is the five periods during seed germination, and the vertical axis is the expression of each time point after normalized fold.

Real-time quantitative reverse transcriptional PCR (qRT-PCR) was used to confirm the expression of 12 representative genes, including 5 upregulated genes and 7 downregulated genes with specific primers (Additional file 7: Table S8). Optimal experiments showed higher amplification efficiency and specificity of 12 genes (Additional file 8: Figure S3). As shown in Figure 9, the expression patterns of these 12 genes were generally consistent with their transcriptional expression models.

## Discussion

### Energy requirements of seed germination

Plants require a large amount of energy for physiological activities during seed germination. Due to the lack of a mineral-uptake system and photosynthetic apparatus, germinating seeds rely on reserve mobilization, mainly starch, proteins and lipids, to provide essential energy for growth until the seedling becomes photoautotrophic [7,38-40]. Wheat is a monocot crop, and its energy reserves are mainly stored in the endosperm, which is degraded for embryo growth during germination. Due to limited oxygen content, energy is supplemented by glycolysis and alcohol fermentation during early seed imbibition. Rate-limiting enzymes PPFK and PK were involved in glycolysis, whereas PDC and ADH were involved in ethanol fermentation. The energy demands of germinating cereal seeds seem to be met mainly by glycolysis [17]. The energy provided by anaerobic respiration cannot satisfy the needs of germinated seeds; at this point, the TCA cycle provides a large amount of energy in oxygen-rich conditions. One of the rate-limiting enzymes in TCA cycle, IDH, accumulates during seed germination and post-germination. The ultimate purpose of starch mobilization, lipid mobilization and protein mobilization is to provide energy. The degradation products of energy reserves always go into glycolysis, then through the TCA cycle and mitochondrial electron transport for ATP synthesis. So, along with the degradation of starch, lipid and protein, the genes related to energy metabolism (glycolysis, TCA cycle, oxidative phosphorylation and mitochondrial electron transport) were initiated from 36 to 48 HAI, indicating the activation of respiratory activities in mitochondrial and ATP release (Figure 5B and Additional file 3: Figure S2B). The above three respiratory pathways are essential for energy provision of a wide range of cellular functions [5,41] as described in this work.

### Starch and sucrose metabolism

The majority of stored endosperm reserves are starch, and their degradation is catalyzed by alpha-amylase. Some genes that are vital to the degradation of starch were found in this study, such as BF293263 and AL820663, which encode alpha-amylase (Table 1). The degradation of starch products can go into glycolysis in two ways. First, amylases can convert the starch into maltose, which then breaks down into glucose and participates in glycolysis; second, starch phosphorylase can convert starch into glucose-1-phosphate, which then enters glycolysis. In this study, most of the genes related to alpha-amylase and starch phosphorylase were upregulated after 12 HAI (Figure 5 and Additional file 2: Table S1). This result indicates that large-scale starch mobilization occurs at about 12 HAI before radicle protrusion, similar to the results found in barley [24]. Pritchard et al. suggested that the majority of sucrose results from storage lipid degradation and not from other soluble sugars within the Arabidopsis seeds [40]; however, our results revealed that initial sucrose is derived from soluble sugars, whereas the majority of sucrose comes from the mobilization of storage starch — lipids as well as some amino acids (Figure 5) — which is similar to germinating barley seeds [24]. Sucrose may be resolved into glucose and fructose by SUSY (sucrose synthase), then enter glycolysis. We found one gene (M26672.1) highly similar to AT3G43190, which encodes SUS4 (Table 1 and Additional file 2: Table S1). SUS4 is one enzyme of SUSY, which is a highly regulated enzyme that reversibly converts sucrose and nucleoside diphosphate into the corresponding nucleoside diphosphate glucose and fructose; this process may occur in most plants, including cereal crops [42-49].

### Protein mobilization

Besides starch, the other most important energy reserves are proteins in the seeds. In early germination, many genes related to protein synthesis, modification and degradation were significantly upregulated (Figure 4). We assume that these proteins are hydrolytic enzymes and proteins relating to hormone signaling, which prepares present and subsequent metabolism. According to the previous analysis, we can also speculate that many genes encode some preceding metabolic enzymes in dry seeds. When the seeds imbibed water, the enzymes activated directly and catalyzed important metabolism processes, such as glycolysis. The protein degradation for the authentic energy requirements occurred in late germination (about 48 HAI); however, the expression of genes encoding some important enzymes in this process, such as cysteine protease, aspartate protease and serine protease, were activated a little early (Figure 4). Germination is known to be partly under the control of gibberellins (GAs), which act to stimulate germination [40], because phytohormone GA plays an important role in triggering the above major proteinases during germination [24,50,51].

### Activation of developmental genes

Shoot and root meristem, as the primary plant meristems, are initiated at opposite poles of the plant embryo. SAM contains a self-renewing population of undifferentiated pluripotent stem cells, which supply cells for the development of all aboveground structures [52,53], whereas RAM produces the primary and lateral root systems [54]. In this study, SAM-related gene CD869453, which belongs to the RNA functional class, is involved in the regulation of transcription and was activated along with seed imbibition. The gene displayed upregulated expression during seed germination and reached its highest expression level at 36 HAI. RAM-related gene CK201893 was rapidly activated after seed imbibition and exhibited an upregulated expression pattern during the seed germination process, which belongs to the redox functional class and is involved in stress response (Table 1). Previous reports have speculated that angiosperm RAM evolved from the SAM, probably due to plants’ adaptation to changing environmental requirements [55,56].

In *Arabidopsis thaliana*, stem cell homeostasis in the SAM is controlled by the negative feedback loop of WUSCHEL-VLAVATA (WUS-CLV) [52,57,58]. This negative feedback was also found in the present study. Both BJ272365 (WUS) and CK202188 (CLV1) had similar expression profiles during seed germination; i.e., they were upregulated from 0 to 24 HAI, downregulated from 24 to 36 HAI, \ then upregulated again at 48 HAI (Table 1). This result suggests a similar developmental pathway during seed germination in wheat as in *Arabidopsis thaliana*.

### Activation of photosynthesis genes

Our results showed that the photosynthetic apparatus was gradually integrated into the seed at about 48 HAI, indicating that subsequent (after 48 HAI) growth depends not only on mobilization of energy reserves, but also on photosynthesis. Genes related to photosynthesis, including light reaction, photorespiration and the Calvin cycle, begin to be largely activated at 48 HAI. Tetrapyrroles have been shown to be important to photosynthesis [59]. As reported in barley [24], during 36 to 48 HAI, the tetrapyrrole synthesis genes as well as photosynthesis genes were activated (Figure 4 and 5). In this period, two genes encoding protochlorophyllide reductase and uroporphyrinogen decarboxylase related to tetrapyrrole synthesis displayed significantly upregulated expression (Figure 4). Photosynthesis uses chlorophylls to convert light to chemical energy, and the protochlorophyllide reductase and uroporphyrinogen decarboxylase are key enzymes that synthesize the chlorophylls; consequently, we can deduce that tetrapyrrole is the intermediate product of chlorophyll synthesis, and the tetrapyrrole could regulate photosynthesis by regulating the chlorophylls.

### Activation of defense genes

Seed development and maturation are accompanied by increased desiccation tolerance [60]. When placed in water, due to the change in external environment, wheat seeds are able to activate a series of mechanisms to respond to many biotic and abiotic stresses during germination (Additional file 9: Figure S4). Cells recognize external stresses and induce many signal proteins, including signaling receptor kinases, light-related proteins, calcium-dependent protein kinases, G-proteins and 14-3-3 proteins (Additional file 10: Figure S5). Studies have found that the tolerance of water, salt, hypoxic, cold stress, and wound or pathogen responses require changes in gene expression [61]. Our results showed that the transcripts of these signal proteins accumulated during germination and that the expression of most genes was upregulated in early germination, thus demonstrating that stress response is mainly regulated by genes. The resumption of respiratory activity, which can be detected within minutes, is one of the first changes upon imbibition [7]. Genes for glutathione peroxidase, redox metabolism and super oxide dismutase, which are involved in scavenging reactive oxygen species (ROS), were reported to be activated during seed germination at 48 HAI in barley [24]. ROS play a pivotal role as triggers of gene expression during biotic and abiotic stresses [49]. In our results, the transcriptional activation of genes for the redox state, peroxidase and glutathione-S-transferase was induced by the respiratory burst. Their expression was largely emerging at about 24 HAI, a little earlier than in barley.

Plant hormones regulate plant growth and mediate responses to both biotic and abiotic stresses [62]. In the hormone signal pathways, besides the participation of two major players (abscisic acid and gibberelin) in controlling germination events, other hormones are involved in an intricate interaction web regulating wheat seed germination [24]. Studies revealed that abscisic acid (ABA) is a positive regulator of dormancy and a negative regulator of germination, whereas gibberelin (GA), ethylene and brassinosteroids (BA) promote seed germination and counteract ABA effects in eudicot seeds [63]. Our results showed that most ABA biosynthetic genes were upregulated slightly from 0 to 12 HAI due to the breaking of dormancy, then downregulated sharply during later germination stages (Additional file 10: Figure S5). However, GA, BA and ethylene biosynthetic genes were largely upregulated from the beginning to the end of wheat seed germination. These results indicate that the regulation of plant hormones appears to be similar in both eudicot and monocotyledon seeds. In addition, the ubiquitin-proteasome pathway has been shown to play a surprisingly important role in hormone signaling [62]. Our results indicated that the ubiquitin-dependent protein degradation started at the very beginning of germination, closely following imbibition (Figure 4). The degradation of these proteins is obviously earlier than that of the storage proteins and starch, which might be a precondition for seed germination, as discovered in rice [17].

Mitogen-activated protein kinases (MAPK) are serine/threonine-specific protein kinases that are activated by a variety of stress stimuli, including osmolarity, drought, temperature, salinity, pathogen infection, wounding and ROS, and modulate cellular activities such as proliferation, gene expression, differentiation, mitosis, cell survival and cell death [64,65]. Studies have reported that most of the substrates for stress-activated MAPKs were transcription factors in animal and yeast cells and plants [66,67]. During wheat seed germination, the expression of many transcription factors (TFs) related to biotic and abiotic stresses changed significantly, such as the ethylene-responsive element binding protein family (ERF TFs), the bZIP transcription factor family (bZIP TFs), the WRKY domain transcription factor family (WRKY domain TFs) and the MYB transcription factor family (MYB TFs). As found in barley [24], these TFs were all upregulated in early seed germination, indicating their essential roles. Related studies showed that the activation of a MAPK can lead to the phosphorylation of transcription factors, which in turn activate gene expression [64]. In tobacco, MAPKs can induce expression of defense genes [68]. MAPKs receive hormonal and other signals and mediate the transcription factors through the MAPK cascade reaction, and then these transcription factors regulate defensive genes encoding stress-related proteins’ responses to external biotic and abiotic stresses. In the current study, the expression of many proteins and pathways related to defense genes, such as cell wall synthesis, beta glucanase PR-proteins, proteolysis and secondary metabolites, were gradually upregulated in early germination, which could protect germinating seeds from the damages of biotic and abiotic stresses (Additional file 9: Figure S4).

## Conclusion

We used transcriptome approaches to characterize gene expression changes through the five stages of seed germination in elite Chinese bread wheat cultivar Jimai 20. Microarray analysis allowed us to detect a large number of genes related to seed germination. The wheat seed germination process included three distinct phases, and a large number of genes involved in seed germination were classified into different functional groups. MapMan and hierarchical cluster analyses yielded a global glance at the changes in gene expression profiles during seed germination. These analyses revealed many important metabolisms (e.g., energy reserves mobilization, the TCA cycle, oxidative phosphorylation, mitochondrial electron transport, photosynthesis and cell wall metabolism) and functional groups (e.g., cellular processes, hormones and signaling, and transporting) involved in seed germination. The co-regulations of the related genes guaranteed regular seed germination. In addition to obvious global changes during seed germination, KEGG and co-expression net analysis revealed the interrelations of many significant filtered differential expression genes, which led to discovery of some pivotal genes and their roles in the seed germination. qRT-PCR analysis further validated the expression patterns of some significant genes. Our transcriptome-level results provide new insights into the thoroughly metabolic changes of seed germination as well as the relationships between some significant genes.

## Methods

### Plant material and seed germination

Elite Chinese bread wheat cultivar (*Triticum aestivum* L.) Jimai 20, with superior gluten quality and high yield performance [34], was used in this study. Seeds with similar sizes and weights were selected as the experimental material. Seeds were washed with distilled water three times and imbibed in distilled water in the dark. After 12 HAI, the seeds were incubated at room temperature. During the germination process, five periods of germination were investigated, including group I (seeds without absorbing water), group II (seeds imbibed water for 12 hours), group III (radicles broken through the episperm about for 24 hours), group IV (one radicle and germ obvious about for 36 hours) and group V (green germ and three radicles visible for about 48 hours). Changes in 1,000-seed weight were tested during five germination stages. Prior to weighing, seeds were paper-blotted for 10 seconds. Samples for morphology and scanning electron microscope (SEM) observation were collected and fixed in different stationary liquids. Collected seeds were stored at -80°C prior to RNA extraction. Three biological replicates for five germination stages were used for microarray hybridization.

### Morphology and SEM observation

The fixed samples were used for morphological observation by stereo microscope. For SEM observation, seeds collected from FAA stationary liquid (50% ethanol 89 ml + glacial acetic acid 6 ml + methanal 5 ml) were fixed with 100 ml for more than 24 hours. Then fixed materials were treated by a series of dehydration solutions (95% ethanol, 100% ethanol, 75% ethanol + 25% isoamyl acetate, 50% ethanol + 50% isoamyl acetate, 25% ethanol + 75% isoamyl acetate, 100% isoamyl acetate, each for 10–20 min). After dehydration, the seeds were treated stepwise for 15 min in mixtures of ethanol and isoamyl acetate with ratios 3:1, 1:1 and 1:3, and finally were soaked in isoamyl acetate for 80 min. After critical point drying, they were observed with a SEM S-4800 FESEM (Hitachi, Japan).

### RNA isolation and microarray hybridization

Total RNA was extracted using prechilled Trizol reagent (Invitrogen, Carlsbad, CA) according to the manufacturer’s directions with some modifications. The RNA was further treated with DNase to remove potential genomic DNA contamination, and integrity and concentration were evaluated using an Agilent 2100 Bioanalyzer (Agilent Technologies, Palo Alto, CA) with 260/280 absorbance ratios of approximately 2.0. Total RNAs were incubated with Oligo dT/T7 primers and reverse-transcribed into double-stranded cDNA. The amplified RNAs were purified and labeled by biotin with Affymetrix’s IVT labeling kit. The biotinylated cDNAs were fragmented and hybridized to the Affymetrix GeneChip® Wheat Genome Array (Affymetrix, Inc., Santa Clara, CA) for 16 hours. After washing and staining, the results were scanned and recorded.

### Data treatments and significant differential gene analysis

The microarray imaging data were analyzed with Microarray suite version 5.0 (Affymetrix Inc.), followed by Spotfire (Spotfire, Somerville, MA). Raw data (CEL files) were normalized at transcript level using the Statistical Algorithm (MAS 5.0 Algorithm). The MAS 5.0 algorithm uses the Tukey’s biweight estimator to provide a robust mean Signal value, the Wilcoxon’s rank test to calculate a significance or p-value and Detection call for each probe set. Background estimation is provided by a weighted average of the lowest 2% of the feature intensities. Mismatch probes are utilized to adjust the perfect match (PM) intensity. Linear scaling of the feature level intensity values, using the trimmed mean, is the default to make the means equal for all analyzed arrays. The MAS 5.0 algorithm (also known as the Statistical Algorithm) analyzes each array independently; as a result, individual probe-specific affinities cannot be considered, and the ability to detect small changes between experiment and control samples is reduced compared with either RMA or PLIER. The primary use of the MAS 5.0 algorithm is to obtain a quick report regarding the performance of the arrays and to identify any obvious problems before submitting the final set of arrays to one of the multichip analysis methods (RMA, PLIER).

The expression of genes adopted a connected loop design for comparative analysis between two time points. Microarrays were performed in triplicate for each time point, and results revealed that the correlation between the replicates for each time point was greater than 0.98. To accurately estimate the per-group gene expression value, differentially expressed probe sets from different germination phases were filtered by ANOVA and corrected by the random variance model (RVM) [69-71]. The gene expression value was the geometric mean of the Robust Multichip Average (RMA) normalized gene signals of 3 samples per time point. The false discovery rate (FDR) was estimated to determine whether certain probe sets were actually significant by repeating the comparison test and the permutation test 1,000 times; FDR aimed to assess the significance of a particular sample having occurred by random chance. The differentially expressed probe sets were selected by two parameters (*p*-value < 0.05 and FDR < 0.05).

STC (Series Test of Cluster) is implemented entirely in Java. The clustering algorithm first selected a set of distinct and representative temporal expression profiles. These model profiles were selected independent of the data. The clustering algorithm then assigned each gene passing the filtering criteria to the model profile that most closely matched the gene’s expression profile as determined by the correlation coefficient. Because the model profiles were selected independent of the data, the algorithm could then determine which profiles had a statistically significant higher number than genes assigned using a permutation test. This test determined an assignment of genes to model profiles using a large number of time point permutations. It then used standard hypothesis testing to determine which model profiles had significantly more genes assigned under the true ordering of time points compared with the average number assigned to the model profile in the permutation runs. Significant model profiles can be analyzed independently or grouped together based on similarity to form clusters of significant profiles [72-74].

Cluster 3.0 was used for hierarchical cluster analysis. MapMan has been shown to be an effective tool to map transcriptome data, define functional categories, and perform time course analyses for identifying significantly overrepresented functional groups. MapMan analysis was mainly based on the barley reference [24]. MapMan BIN was inferred based on both automatic and manual annotations.

The microarray data used in this study have been deposited in the NCBI GEO database and are accessible through GEO Series accession number GSE49821 (http://www.ncbi.nlm.nih.gov/geo/query/acc.cgi?acc = GSE49821).

### Signal-net analysis

Signal network maps were constructed by Java in the KEGG database. If there was confirmative evidence that two genes interacted with each other, an interaction edge was assigned between them [75-77]. In the networks, the nodes were mainly genes (protein, compound, etc.) and edges representing relation types between the nodes; e.g., activation or phosphorylation. Degree was defined as the sum of connection strengths with the other network genes: $K_{i}=\sum_{u\neq i}a_{\mathrm{ui}}$. For a gene in the network, the number of source genes of a gene was called the indegree of the gene, and the number of target genes of a gene was its outdegree. After each gene was ranked according to its degree from high to low, the network focal points represented the genes that had the highest value of degree. These genes had the greatest impact on the structure of the whole network and the relationships among other genes.

### Pathway and path-net analysis

Similarly, pathway analysis was used to find out the significant pathways of the differentially expressed genes according to the KEGG, Biocarta and Reatome databases. Still, the two-side Fisher’s exact test and *x*^2^test were used to classify the significant pathways, and the threshold of significance was defined by *p*-value < 0.05 and FDR < 0.05. The FDR value was used to correct the *p*-value [78-80].

The Path-Net was the interaction net of the significant pathways of the differential expression genes and was built according to the interaction among pathways of the KEGG database to find the interaction among the significant pathways directly and systemically. It could summarize the pathway interaction of differential expression genes and determine why a certain pathway was activated [79].

### Construction and topological attributes of co-expression networks

In biological processes, gene co-expression networks can be constructed from functional gene associations to identify gene interaction. For each pair of genes, we calculated the Pearson correlation and chose significant correlation pairs with which to construct the network [81]. In the network, cycle nodes represent genes, and edges between two nodes represent interactions between genes. The purpose of network structure analysis is to locate core regulatory factors (genes) that connect most adjacent genes and have the highest k-core values, or degrees values. A k-core of a network is a sub-network in which all nodes are connected to at least k other genes in the sub-network. As a result, the rank of a k-core value describes the complexity of the gene association relationship. The maximum core order is termed the main core or the highest k-core of the graph [82]. Cycles with identical colors are part of the same subgraph [83,84]; moreover, to study various properties of networks, degree centrality is also an important measure of gene centrality within a network built to determine relative importance. Degree centrality is defined as the link numbers one node has to another.

### Quantitative real-time PCR (qRT-PCR)

Differential expression genes were verified by qRT-PCR. After RNA isolation, first-strand cDNA was synthesized in a 20-μl volume containing 0.5 μl AMV reverse transcriptase (Promega), 0.5 μl RNase inhibitor (Promega), 1 μl oligo dT primer, 2 μl dNTP mixture, 4 μl MgCl2 (25 mM), 2 μl 10× reverse transcriptase buffer and 4 μl RNA sample. The reaction mixture was incubated at 42°C for 60 min.

A double standard curve method was used to detect gene expression levels. ADP-ribosylation factor was used as the internal control, which was identified as one of the most stably expressed genes [85]. Gene-specific primers were designed using Primer 5.0, and their specificities were checked by the melting curves of the RT-PCR products. Each qRT-PCR reaction was performed in 20-μl volumes containing 10 μl 2 × SYBR Premix Ex Taq (TaKaRa), 2 μl 50-fold diluted cDNA, 0.4 μl of each gene-specific primer, and 7.2 μl ddH2O. PCR conditions were as follows: 95°C for 3 min, 45 cycles of 15 sec at 95°C, 57°C for 15 sec and 72°C for 20 sec. Three replicates were used for each sample. Reactions were conducted in a CFX96 Real-Time PCR Detection System (Bio-Rad). All data were analyzed with CFX Manager Software (Bio-Rad).

## Abbreviations

ABA: Abscisic acid; ADH: Alcohol dehydrogenase; ALDH: Aldehyde dehydrogenase; BA: Brassinosteroids; FA: Fatty acid; FDR: False Discovery Rate; GA: Gibberelin; HAI: Hour after imbibition; HCA: Hierarchical cluster analysis; IDH: Isocitrate dehydrogenase; KEGG: Kyoto Encyclopedia of Genes and Genomes; MAPK: Mitogen-activated protein kinases; PDC: Pyruvate decarboxylase; PDH: Pyruvate dehydrogenase; PPFK: Phosphofructokinase; PK: Pyruvate kinase; qRT-PCR: Real-time quantitative reverse transcriptional PCR; RMA: Robust Multichip Average; RVM: Randomized Variance Model; RAM: Root apical meristem; ROS: Reactive oxygen species; SEM: Scanning Electronic Microscope; SAM: Shoot apical meristem; SUSY: Sucrose synthase; STC: Series Test of Cluster; TF: Transcription Factor; UGPase: UDP-glucose pyrophosphorylase.

## Competing interests

This manuscript has no financial or non-financial competing interests.

## Authors’ contributions

YY and GG carried out all experiments and data analysis. LD and HY performed the preparation of RNA, cDNA, qRT-PCR and bioinformatics analyses. JL helped English writing and proofreading of the manuscript. LX and YY conceived the study, planned experiments, and helped draft the manuscript. All authors read and approved the final manuscript.

## Supplementary Material

Additional file 1: Figure S1 The diameter changes of A-type starch granules during five seed germination stages. The horizontal axis is seed germination periods, and the vertical axis is changes in diameter. Click here for file

Additional file 2: Table S1 Complete list of normalized expression values obtained from three experimental series based on independently grown plant material. Table S2. Overview of MapMan functional classes (BINs and subBINs) of the Affymetrix GeneChip® Wheat Genome Array. Table S3. Complete list of differentially expressed genes obtained from the whole list of normalized expression values based on *p*-value and FDR. Click here for file

Additional file 3: Figure S2 MapMan metabolism overview maps showing differences in transcript levels (12 versus 24 HAI and 36 versus 48 HAI) during seed germination (for further details, see legend to Figure 5). Click here for file

Additional file 4: Table S4 The genes list for the construction of signal network. Table S5. The complete list of rice gene sequence after blast base on the differential genes or EST sequence of wheat. Click here for file

Additional file 5: Table S6 The significant pathways list of pathway-net. Click here for file

Additional file 6: Table S7 The complete list of co-expression genes. Click here for file

Additional file 7: Table S8 Primer sequences used for quantitative RT-PCR (qRT-PCR). Click here for file

Additional file 8: Figure S3 The standard curve and melt peak of genes. Click here for file

Additional file 9: Figure S4 Mapman displays the expression of genes related with regulation and response to external environment between 12 HAI and 24 HAI during seed germination with some modification. Click here for file

Additional file 10: Figure S5 Mapman displays the regulation overview maps showing differences in transcript levels between 0 and 48 HAI during seed germination. On the logarithmic color scale, blue represents downregulated transcripts, and red represents upregulated transcripts. Click here for file
