# Supplementary figures and images for: Transcriptome analysis during seed germination of elite Chinese bread wheat cultivar Jimai 20
Source: BMC Plant Biol. 2014 Jan 13;14:20. doi: 10.1186/1471-2229-14-20 (PMC3923396; doi:10.1186/1471-2229-14-20)

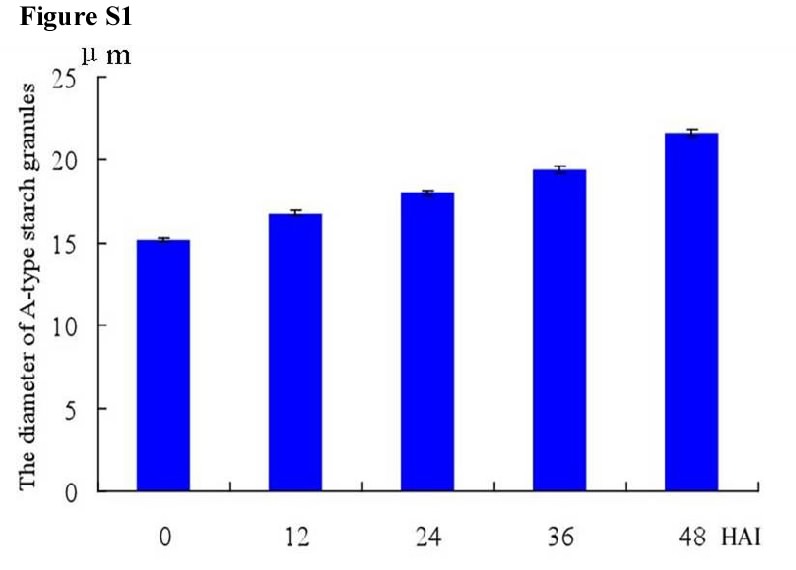

Supplement: Additional file 1: Figure S1 — The diameter changes of A-type starch granules during five seed germination stages. The horizontal axis is seed germination periods, and the vertical axis is changes in diameter. [file 1471-2229-14-20-S1.jpeg]

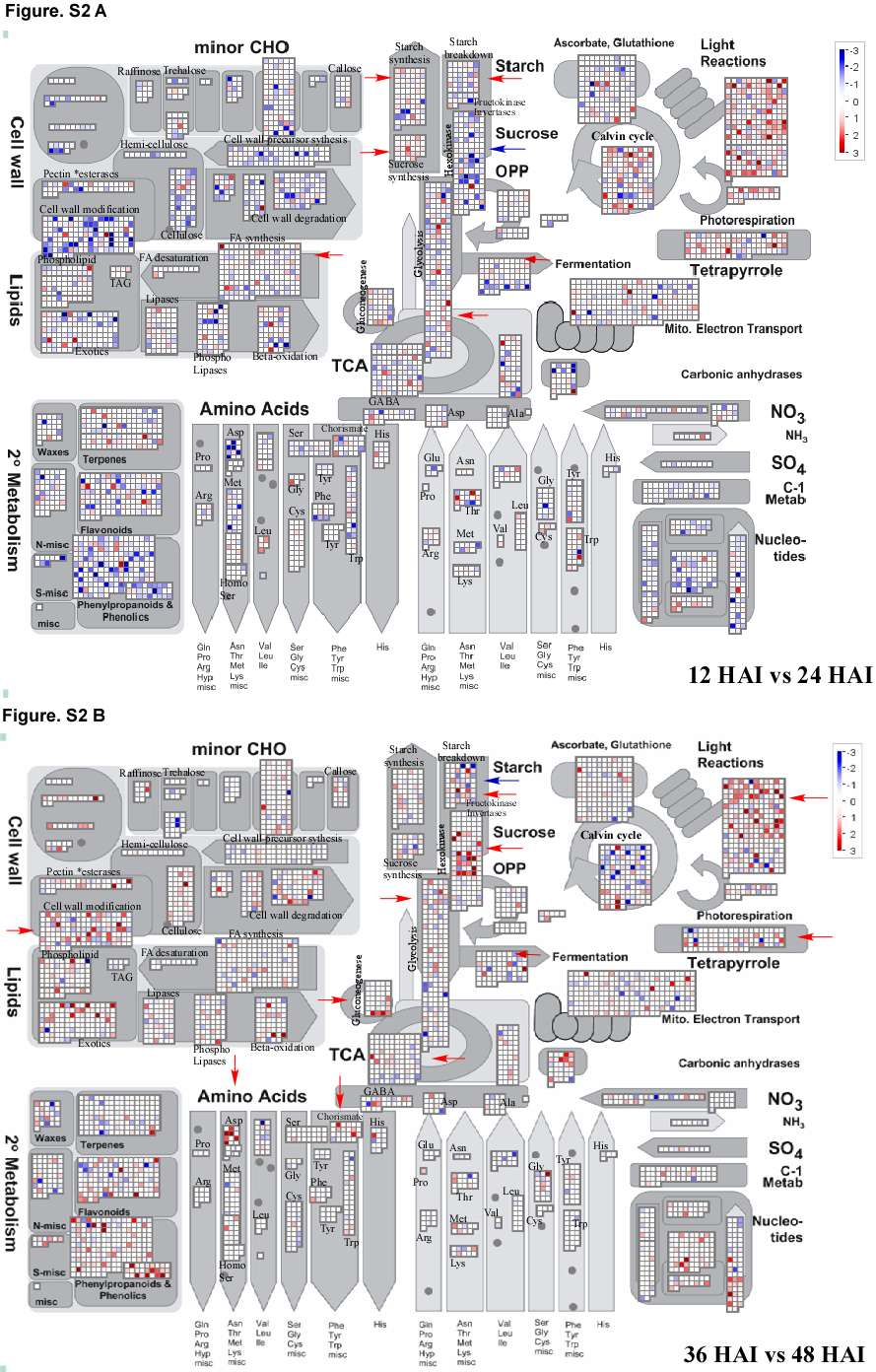

Supplement: Additional file 3: Figure S2 — MapMan metabolism overview maps showing differences in transcript levels (12 versus 24 HAI and 36 versus 48 HAI) during seed germination (for further details, see legend to Figure 5). [file 1471-2229-14-20-S3.tiff]

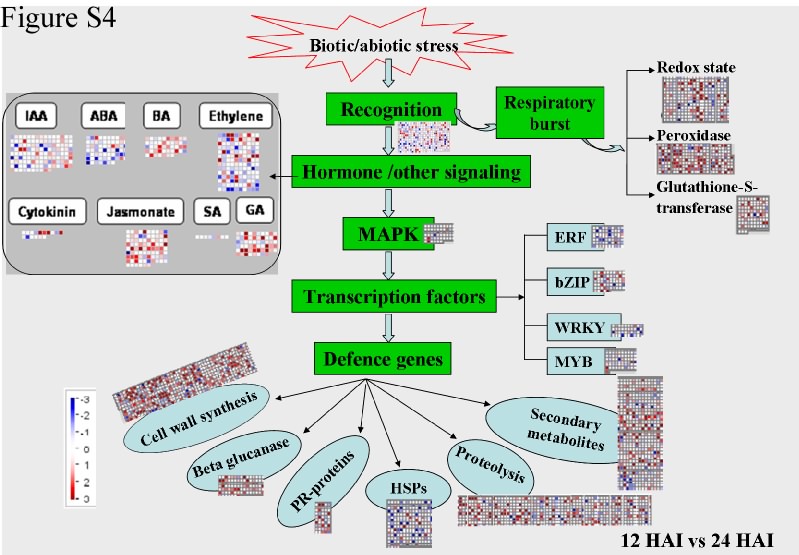

Supplement: Additional file 9: Figure S4 — Mapman displays the expression of genes related with regulation and response to external environment between 12 HAI and 24 HAI during seed germination with some modification. [file 1471-2229-14-20-S9.jpeg]

Figure S5

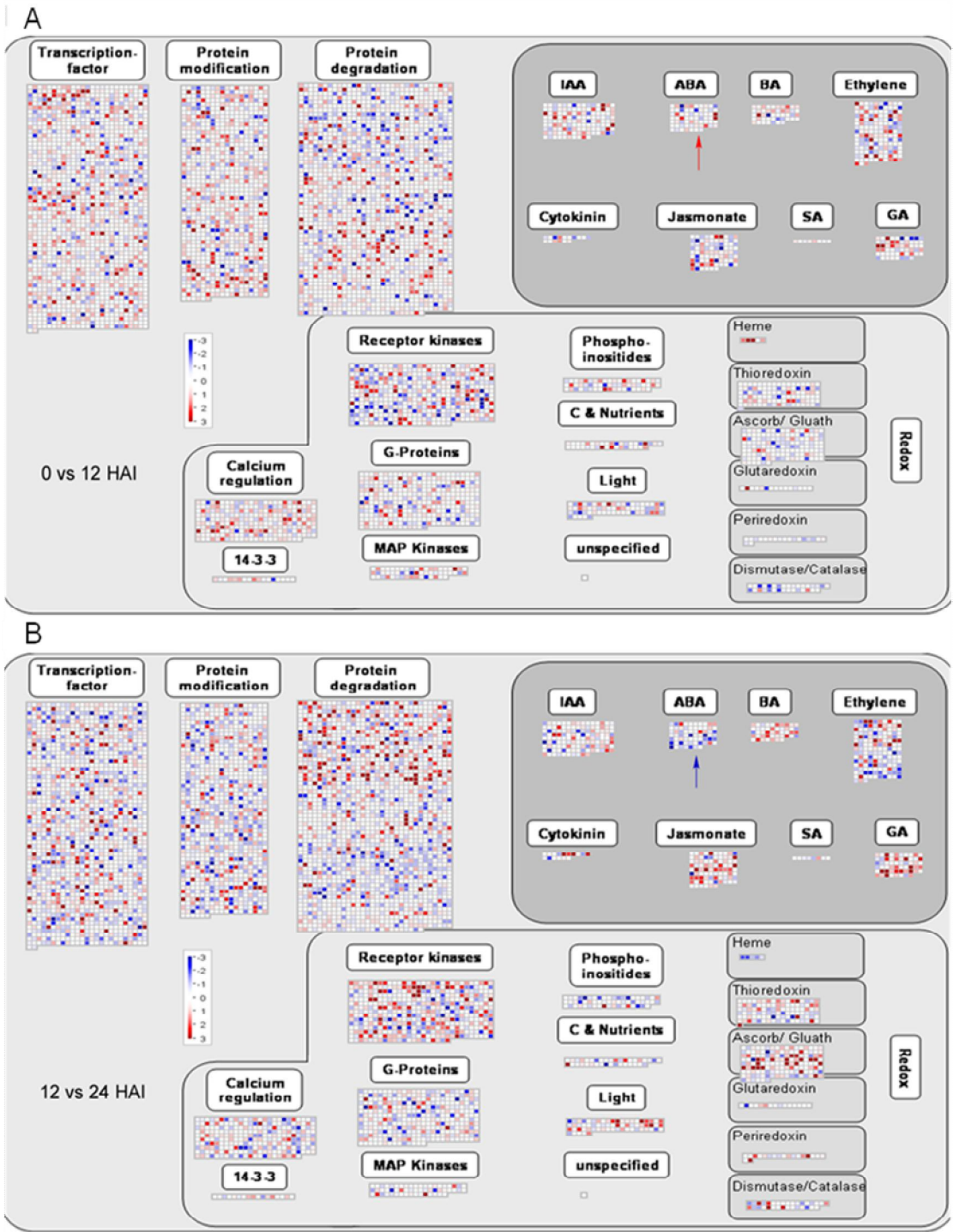

C

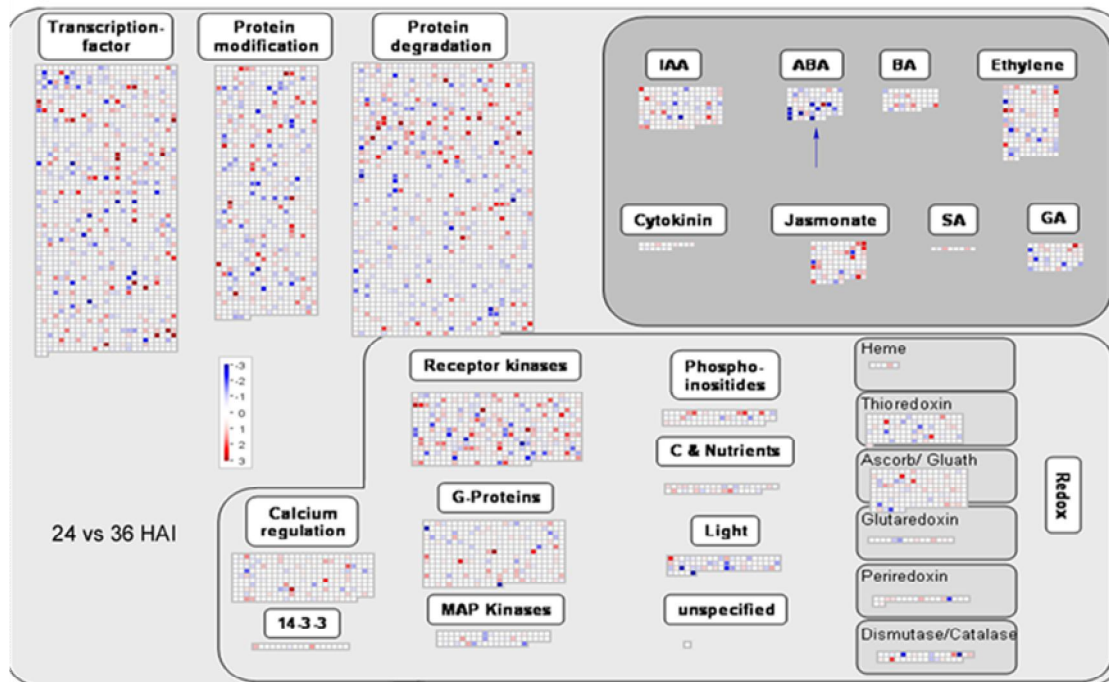

D

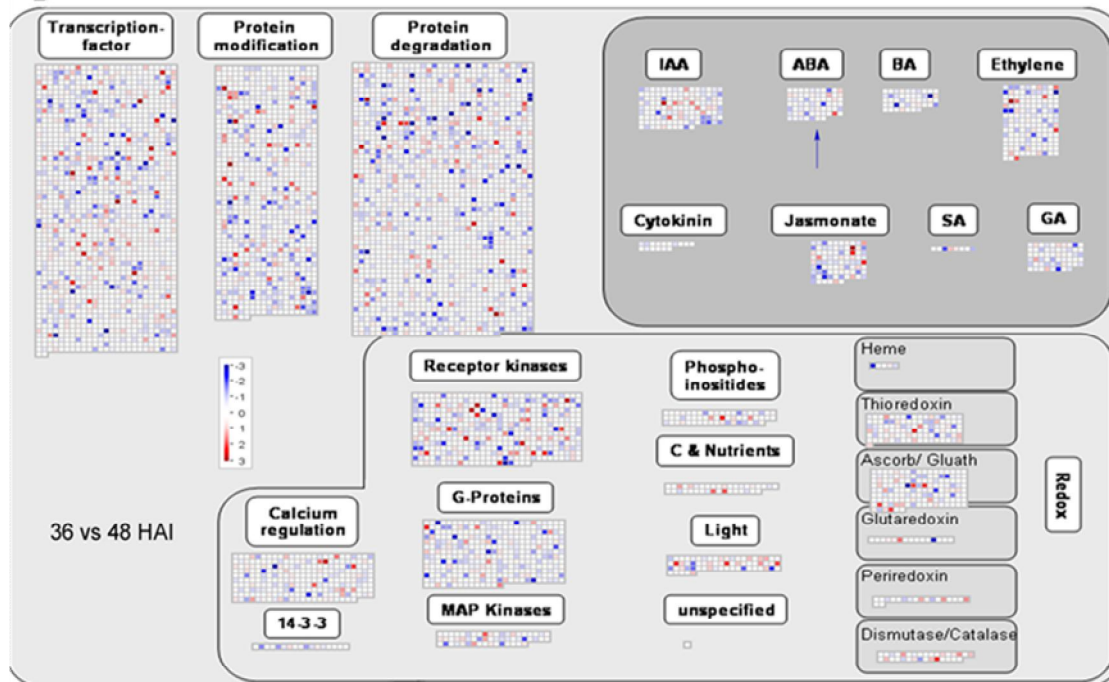

Supplement: Additional file 10: Figure S5 — Mapman displays the regulation overview maps showing differences in transcript levels between 0 and 48 HAI during seed germination. On the logarithmic color scale, blue represents downregulated transcripts, and red represents upregulated transcripts. [file 1471-2229-14-20-S10.pdf]
