# Supplementary material for: Transcriptome analysis during seed germination of elite Chinese bread wheat cultivar Jimai 20
Source: BMC Plant Biol. 2014 Jan 13;14:20. doi: 10.1186/1471-2229-14-20 (PMC3923396; doi:10.1186/1471-2229-14-20)
Supplement: Additional file 8: Figure S3 — The standard curve and melt peak of genes. [file 1471-2229-14-20-S8.pdf]

Figure S3

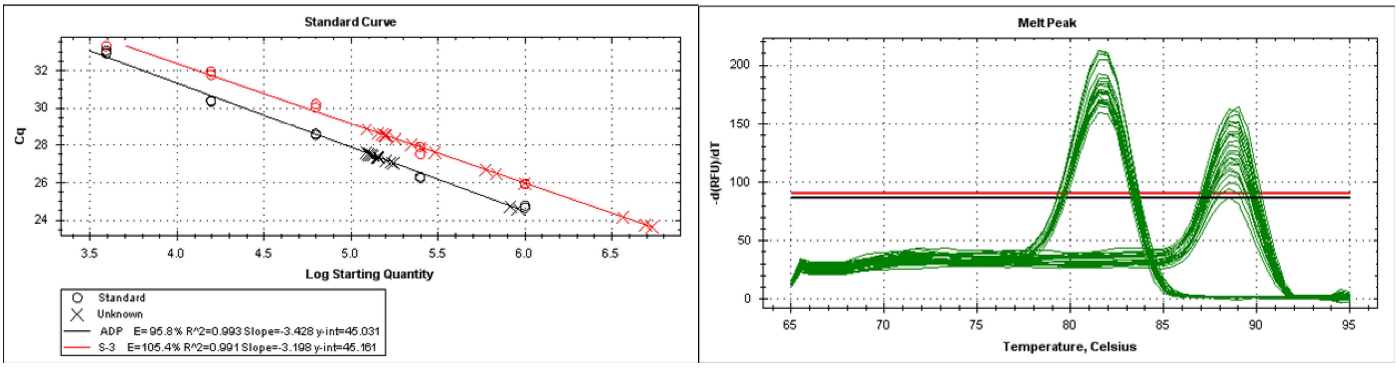

UTP-glucose-1-phosphate uridylyltransferase

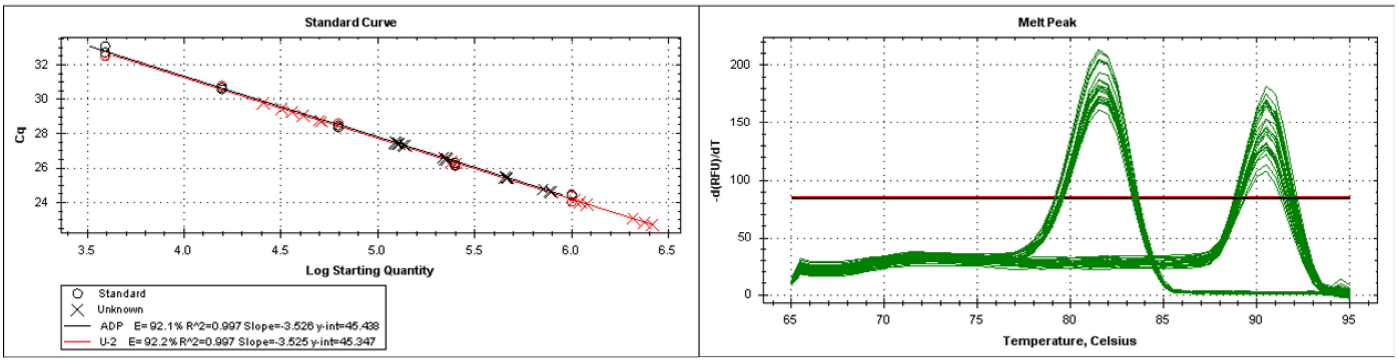

Hypothetical protein

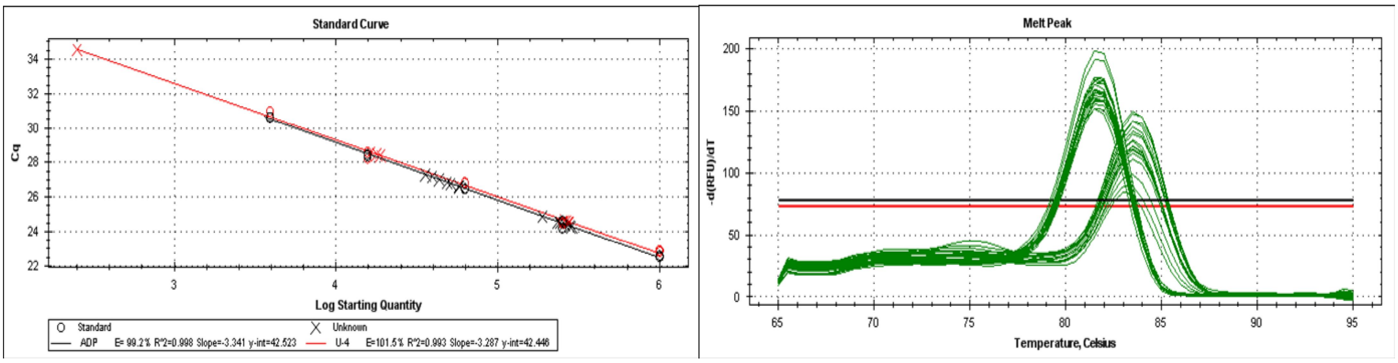

Histone H4

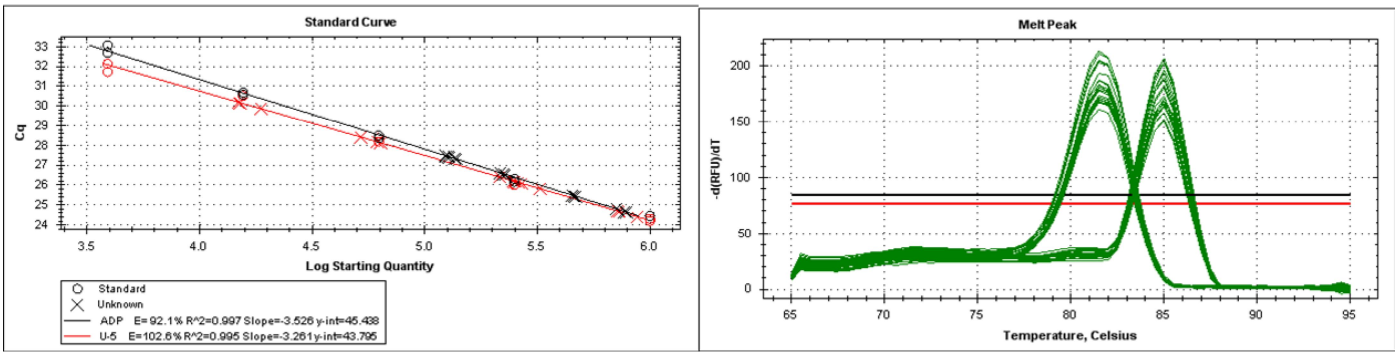

T-complex protein 1, delta subunit

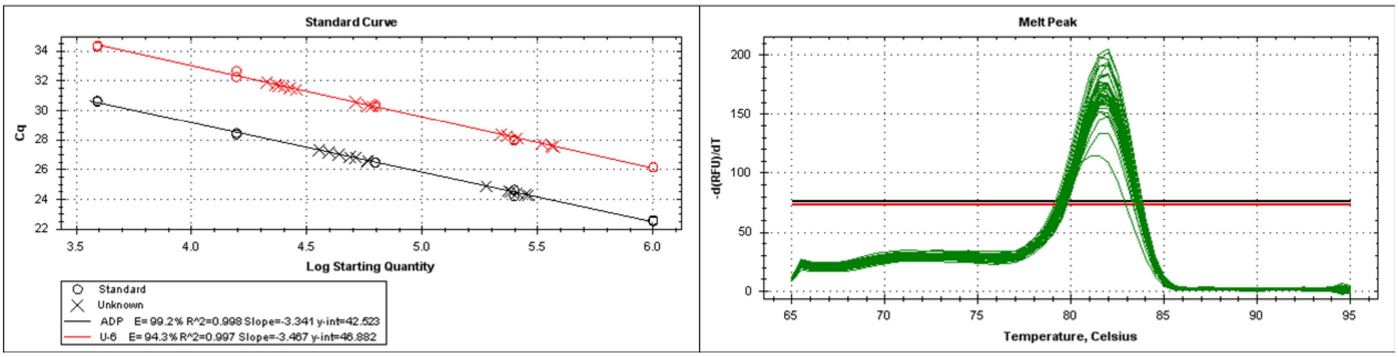

Ribosomal protein L13 containing protein

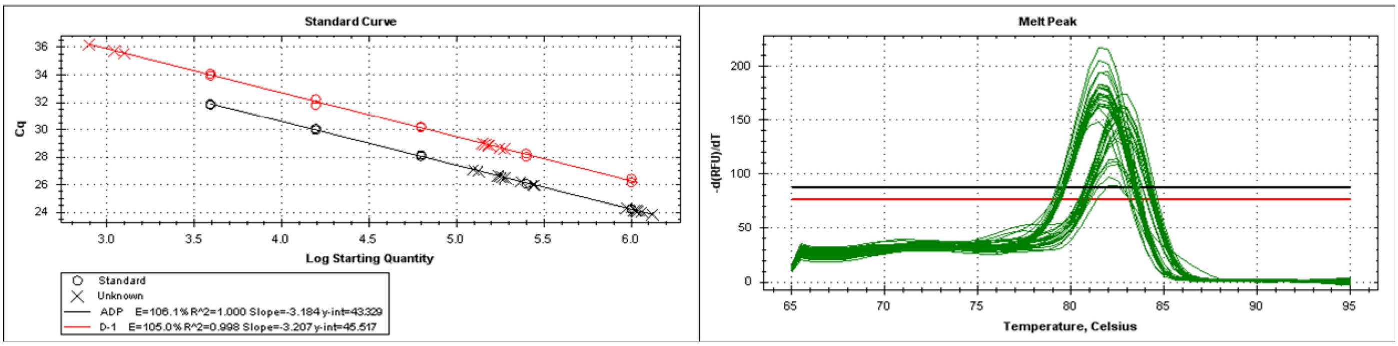

Embryonic abundant protein 1

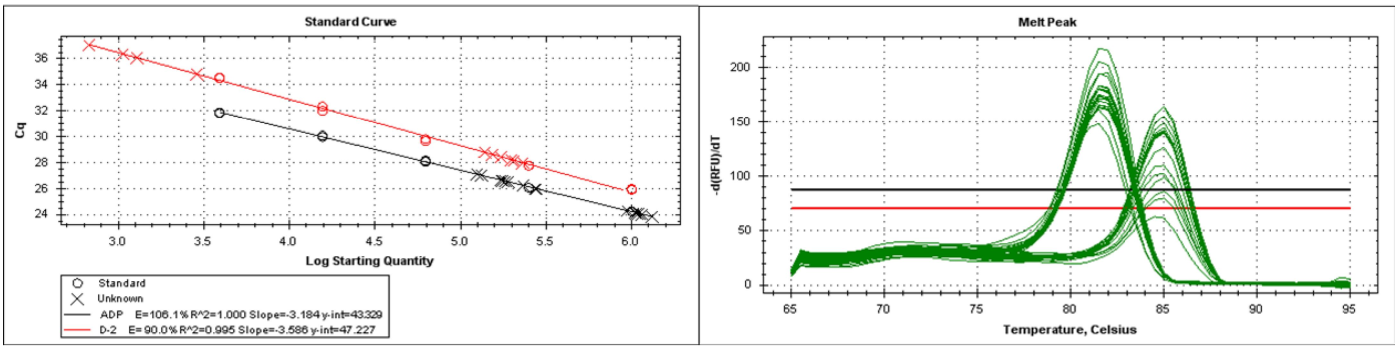

Vacuolar cation/proton exchanger 1b

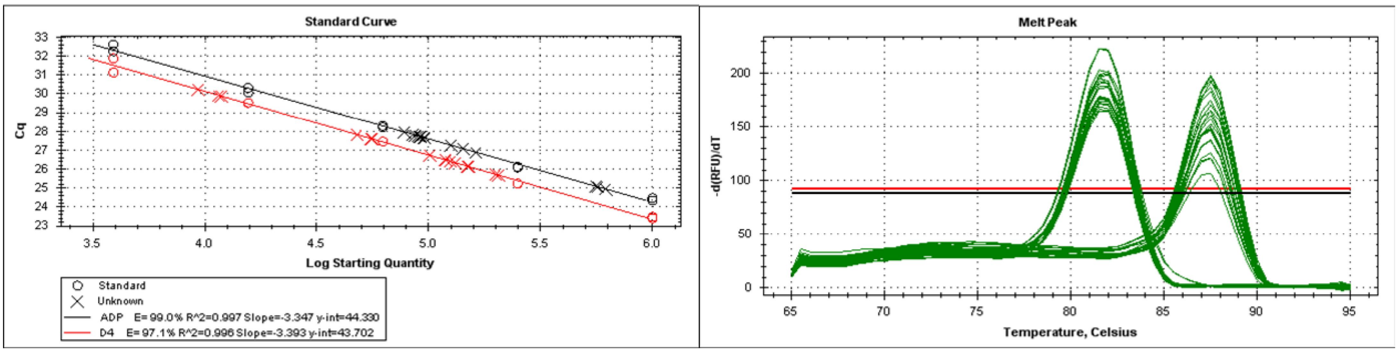

Late embryogenesis abundant protein Lea14-A

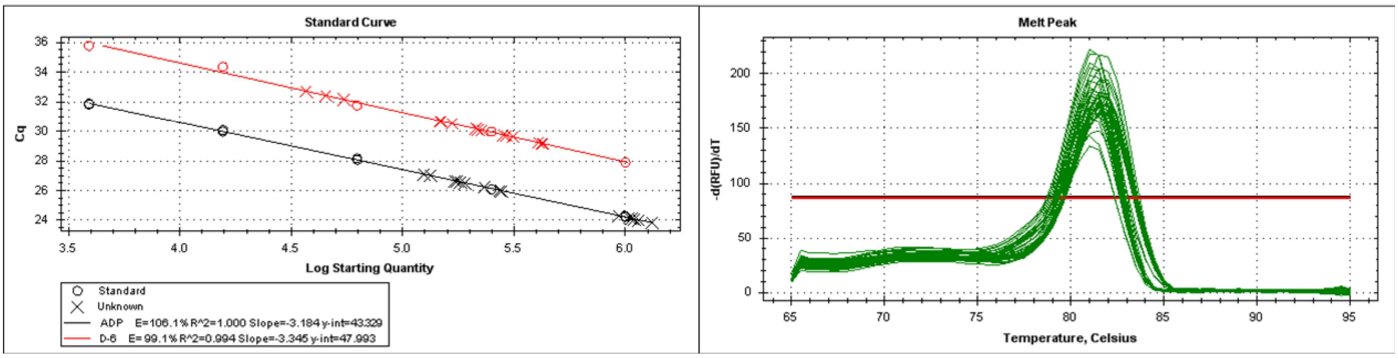

Aldehyde dehydrogenase family protein

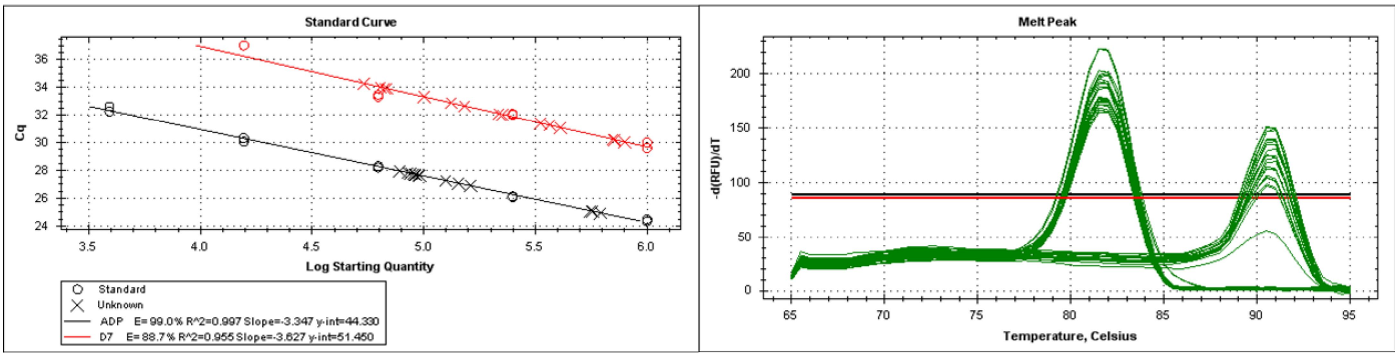

AP2 domain containing protein

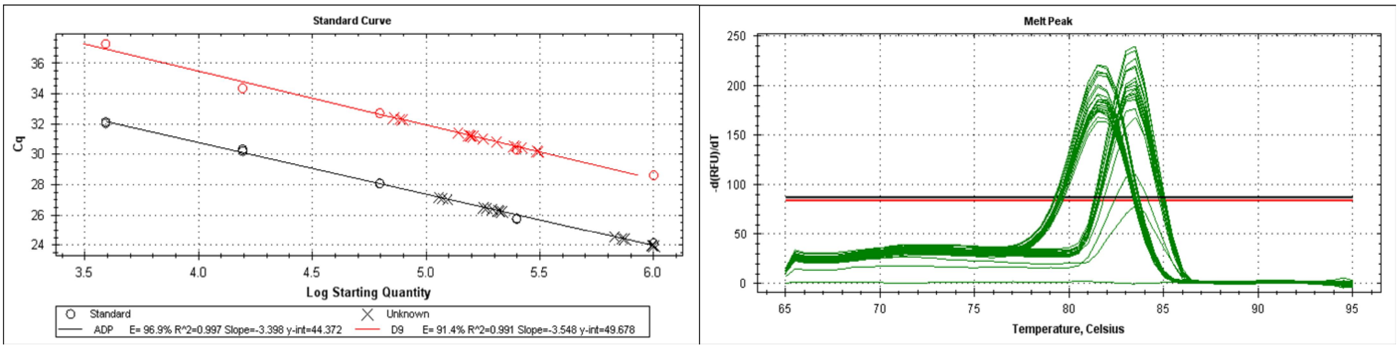

NAD dependent epimerase or dehydratase family protein

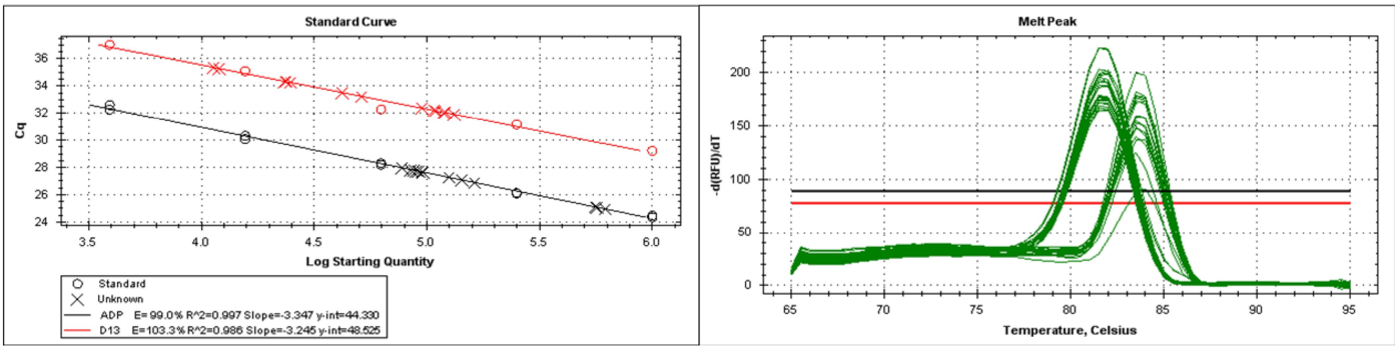

Polyubiquitin containing 7 ubiquitin monomers
